# Supplementary material for: Modeling glioblastoma heterogeneity as a dynamic network of cell states
Source: Mol Syst Biol. 2021 Sep 16;17(9):e10105. doi: 10.15252/msb.202010105 (PMC8444284; doi:10.15252/msb.202010105)
Supplement: Supplementary file 6 — Source Data for Figure 5 [file MSB-17-e10105-s004.zip › Figure5A_sourcedata/GSEA_3071/nosigdb_stateVI.GseaPreranked.1622795169056/NB_KI_CLUSTER15.html]

Details for gene set NB\_KI\_CLUSTER15[GSEA]

|  || Dataset | state63071 |
| Phenotype | NoPhenotypeAvailable |
| Upregulated in class | na\_pos |
| GeneSet | NB\_KI\_CLUSTER15 |
| Enrichment Score (ES) | 0.18125725 |
| Normalized Enrichment Score (NES) | 1.2693142 |
| Nominal p-value | 0.13375796 |
| FDR q-value | 0.21862504 |
| FWER p-Value | 0.98 |
Table: GSEA Results Summary

  

Fig 1: Enrichment plot: NB\_KI\_CLUSTER15      
 Profile of the Running ES Score & Positions of GeneSet Members on the Rank Ordered List

  

| PROBE | GENE SYMBOL | GENE\_TITLE | RANK IN GENE LIST | RANK METRIC SCORE | RUNNING ES | CORE ENRICHMENT || 1 | KRT8 |  |  | 42 | 0.895 | 0.0182 | Yes |
| 2 | SOCS2 |  |  | 72 | 0.803 | 0.0369 | Yes |
| 3 | HSBP1L1 |  |  | 104 | 0.735 | 0.0528 | Yes |
| 4 | CHCHD7 |  |  | 198 | 0.604 | 0.0469 | Yes |
| 5 | STX3 |  |  | 228 | 0.582 | 0.0582 | Yes |
| 6 | MAN1A1 |  |  | 250 | 0.563 | 0.0712 | Yes |
| 7 | SYNJ2 |  |  | 270 | 0.555 | 0.0844 | Yes |
| 8 | NCEH1 |  |  | 351 | 0.512 | 0.0791 | Yes |
| 9 | ATF5 |  |  | 401 | 0.490 | 0.0817 | Yes |
| 10 | MAST4 |  |  | 446 | 0.473 | 0.0852 | Yes |
| 11 | EHD1 |  |  | 455 | 0.469 | 0.0987 | Yes |
| 12 | KIFC3 |  |  | 484 | 0.460 | 0.1062 | Yes |
| 13 | MAP2K3 |  |  | 550 | 0.440 | 0.1027 | Yes |
| 14 | EFHD2 |  |  | 598 | 0.425 | 0.1038 | Yes |
| 15 | KCNK6 |  |  | 611 | 0.422 | 0.1145 | Yes |
| 16 | TBC1D4 |  |  | 652 | 0.411 | 0.1171 | Yes |
| 17 | KIF1C |  |  | 696 | 0.399 | 0.1184 | Yes |
| 18 | SUPT3H |  |  | 710 | 0.395 | 0.1279 | Yes |
| 19 | SLC35E4 |  |  | 792 | 0.378 | 0.1179 | Yes |
| 20 | LRRN4CL |  |  | 794 | 0.378 | 0.1302 | Yes |
| 21 | FKBP14 |  |  | 813 | 0.374 | 0.1377 | Yes |
| 22 | UBASH3B |  |  | 820 | 0.373 | 0.1485 | Yes |
| 23 | RNF135 |  |  | 842 | 0.369 | 0.1549 | Yes |
| 24 | PML |  |  | 874 | 0.365 | 0.1585 | Yes |
| 25 | ACOT9 |  |  | 906 | 0.360 | 0.1618 | Yes |
| 26 | AP1S3 |  |  | 910 | 0.359 | 0.1730 | Yes |
| 27 | RPS6KA4 |  |  | 924 | 0.357 | 0.1813 | Yes |
| 28 | MSL3 |  |  | 992 | 0.347 | 0.1741 | No |
| 29 | FHL3 |  |  | 1065 | 0.335 | 0.1652 | No |
| 30 | C19orf24 |  |  | 1173 | 0.320 | 0.1459 | No |
| 31 | TMEM150A |  |  | 1216 | 0.313 | 0.1446 | No |
| 32 | DAPK3 |  |  | 1298 | 0.303 | 0.1320 | No |
| 33 | CFAP300 |  |  | 1381 | 0.294 | 0.1189 | No |
| 34 | TTC27 |  |  | 1415 | 0.290 | 0.1194 | No |
| 35 | DSE |  |  | 1484 | 0.282 | 0.1097 | No |
| 36 | PER2 |  |  | 1488 | 0.281 | 0.1183 | No |
| 37 | SP140L |  |  | 1536 | 0.275 | 0.1143 | No |
| 38 | SESN2 |  |  | 1541 | 0.275 | 0.1224 | No |
| 39 | DNMBP |  |  | 1596 | 0.269 | 0.1163 | No |
| 40 | FAM13A |  |  | 1611 | 0.268 | 0.1213 | No |
| 41 | RFLNB |  |  | 1672 | 0.263 | 0.1133 | No |
| 42 | GALNT7 |  |  | 1674 | 0.262 | 0.1218 | No |
| 43 | ENPP1 |  |  | 1677 | 0.262 | 0.1300 | No |
| 44 | SEMA7A |  |  | 1696 | 0.260 | 0.1336 | No |
| 45 | FURIN |  |  | 1702 | 0.260 | 0.1409 | No |
| 46 | NCK2 |  |  | 1865 | -0.260 | 0.1042 | No |
| 47 | LRCH1 |  |  | 1869 | -0.260 | 0.1121 | No |
| 48 | MOB3B |  |  | 1883 | -0.261 | 0.1172 | No |
| 49 | LONRF1 |  |  | 1954 | -0.271 | 0.1067 | No |
| 50 | RFFL |  |  | 1963 | -0.273 | 0.1135 | No |
| 51 | FMNL3 |  |  | 1992 | -0.277 | 0.1150 | No |
| 52 | STX17 |  |  | 2017 | -0.281 | 0.1176 | No |
| 53 | DPY19L1 |  |  | 2046 | -0.284 | 0.1193 | No |
| 54 | FAM84B |  |  | 2080 | -0.291 | 0.1198 | No |
| 55 | MARCH2 |  |  | 2187 | -0.313 | 0.1006 | No |
| 56 | ZNRF2 |  |  | 2200 | -0.314 | 0.1077 | No |
| 57 | SPATA13 |  |  | 2300 | -0.333 | 0.0911 | No |
| 58 | STAT2 |  |  | 2345 | -0.342 | 0.0902 | No |
| 59 | ALG6 |  |  | 2401 | -0.353 | 0.0866 | No |
| 60 | TSPAN14 |  |  | 2676 | -0.418 | 0.0239 | No |
| 61 | ST8SIA4 |  |  | 2719 | -0.430 | 0.0265 | No |
| 62 | LDLRAD4 |  |  | 2854 | -0.478 | 0.0049 | No |
| 63 | GNS |  |  | 2973 | -0.525 | -0.0106 | No |
| 64 | MIRLET7BHG |  |  | 3042 | -0.559 | -0.0109 | No |
| 65 | CDH13 |  |  | 3410 | -0.913 | -0.0832 | No |
| 66 | SEL1L3 |  |  | 3549 | -1.336 | -0.0771 | No |
| 67 | FGD4 |  |  | 3563 | -1.431 | -0.0329 | No |
| 68 | MAP3K1 |  |  | 3584 | -1.605 | 0.0151 | No |
Table: GSEA details [plain text format]

  

Fig 2: NB\_KI\_CLUSTER15: Random ES distribution      
 Gene set null distribution of ES for **NB\_KI\_CLUSTER15**
